# Association of *Ovocalyxin-32* Gene Variants with Egg Quality Traits in Indigenous Chicken Breeds

**DOI:** 10.3390/ani14203010

**Published:** 2024-10-17

**Authors:** Haitham A. Yacoub, Moataz M. Fathi, Ibrahim H. Al-Homidan, Moataz I. Badawy, Mohamed H. Abdelfattah, Mohamed F. Elzarei, Osama K. Abou-Emera, Gamal N. Rayan

**Affiliations:** 1 Department of Cell Biology, Biotechnology Research Institute, National Research Centre, Giza 12622, Egypt; 2 Department of Animal and Poultry Production, College of Agriculture and Food, Qassim University, Al-Qassim 51452, Saudi Arabia; mmfathi@fulbrightmail.org (M.M.F.); homidani@yahoo.com (I.H.A.-H.); zray@qu.edu.sa (M.F.E.); osama_emera@yahoo.com (O.K.A.-E.); 3 Department of Poultry Production, Faculty of Agriculture, Ain Shams University, Hadayek Shoubra, Cairo 11241, Egypt; gahmed@kfu.edu.sa; 4 Department of Biotechnology, Animal Production Research Institute, Agricultural Research Center, Dokki, Giza 12618, Egypt; motaz.badwyyy@gmail.com; 5 Department of Poultry Breeding Research, Animal Production Research Institute, Agricultural Research Center, Dokki, Giza 12618, Egypt; 6 Department of Animal Production, Faculty of Agriculture, Suez Canal University, Ismailia 41522, Egypt; 7 Department of Animal and Fish Production, College of Agricultural and Food Sciences, King Faisal University, Al-Ahsa 31982, Saudi Arabia

**Keywords:** native chicken, *ovocalyxin-32* gene, SNP, egg quality

## Abstract

This study sought to evaluate the genetic variations of the *ovocalyxin-32* gene and its association with egg quality traits in indigenous chicken populations, focusing on exons 1 and 6. Genotype frequencies of SNPs (G/T and A/G) within these exons were assessed for their conformity to the Hardy–Weinberg equilibrium (HWE) across several strains. While most strains exhibited close adherence to HWE expectations, some like light-brown and gray strains indicated substantial discrepancies, particularly for the TT genotype, which points towards the possible effects of genetic drift as well as selection pressures. This study also analyzed the influence of such SNPs on egg quality parameters. A thinner eggshell, reduced shell weight, and decreased breaking strength were associated with the G/T SNP in exon 1, suggesting a likely negative effect on egg quality in T allele carriers. Conversely, the AG genotype displayed better performance in shell thickness, weight and egg weight in the A/G SNP in exon 1, whilst yolk height was best improved by the AA genotype compared to breaking strength. For instance, in exon 6, the A/G SNP enhanced the shell and yolk quality among AG genotypes, while the CC genotype resulted in better eggshell characteristics with enlarged yolks because the C/T SNP was linked. Nonetheless, there were no significant deviations from the HWE despite these associations, which suggested that most breeds had a stable genetic background. Further, considering SNPs’ additive and dominant effects in this research, it was indicated that additive effects account for phenotypic expressions given by the G/T SNP located at exon 1. In contrast, significant additive and dominant effects were observed under the A/G SNP situated at the exon. Generally, it therefore could be concluded from this study that specific SNPs within the *ovocalyxin-32* gene may act as good markers for marker-assisted selection (MAS) that can improve desired characteristics—such as those of egg quality—in indigenous chicken breeds. This study demonstrated that both additive and dominance effects must be taken into account when performing genetic analyses, thereby emphasizing the complexity of phenotypic variation caused by genetic mechanisms in native chicken races.

## Simple Summary

The genetic polymorphisms in the *ovocalyxin-32* gene were studied in the present work with regard to egg quality traits in indigenous chicken populations. In the same investigation, based on exons 1 and 6, the frequencies of G/T and A/G SNPs regarding the Hardy–Weinberg equilibrium (HWE) and their role in egg quality were determined. The T allele of the G/T SNP in exon 1 was associated with thinner shells and lower shell strength, while the A/G SNP in exon 1 resulted in eternal eggs with thicker shells in AG and AA genotypes. Regarding this, the A/G SNP in exon 6 was able to enhance shell and yolk quality in AG genotypes. These SNPs could be considered candidate genetic markers for marker-assisted selection (MAS) focusing on egg quality improvement, which has additive and dominance genetic effects on phenotypic variation.

## 1. Introduction

Recently, attention has been increasingly paid to research concerning the genetic bases responsible for the main economic traits of poultry, e.g., those that affect egg quality. The *ovocalyxin-32* (*OCX-32*) gene has emerged as a major target due to its role in the composition of the shell and other egg quality traits [1,2]. Primarily expressed in the eggshell gland, the *OCX-32* gene imparts to this structure its form and integrity [3]. Various investigators have hypothesized that eggs’ SNPs (single nucleotide polymorphisms) within this gene may be important in determining various aspects of egg quality, making this an interesting subject for breeding and selection scenarios [4,5].

Knowledge regarding the genetics underlying economically important traits such as egg quality is critical for the improvement of breeding schemes as well as sustainability maintenance for native chicken breeds. Other studies have shown that specific SNPs occurring at the exon-1 and exon-6 regions of *OCX-32* contribute heavily to shell thickness, the weight of eggs, and Haugh units, among other aspects, which are key elements indicating internal qualities in eggs [6,7].

The Hardy–Weinberg equilibrium is a theoretical framework that explains the distribution of genotypes expected in an ideal population where there are no mutations, migrations and/or other such factors affecting allele frequencies [8,9]. Therefore, this study will conduct genetic analysis through the Hardy–Weinberg equilibrium (HWE) to understand how various SNPs situated across *OCX-32* correlate with egg quality traits among native chicken strains. The actual links between these SNPs and egg quality traits indicated that this research focused on genes underlying economically important characteristics [10,11,12]. Concerning the native chicken breeds, we checked whether the observed genotype and allele frequencies at the *OCX-32* gene were consistent with HDW predictions, helping to reveal forces acting upon genetic variation in these populations [13,14].

Variations in the distribution of genotypes and alleles at different SNP loci of the *OCX-32* gene across different breeds reflect the unique selection pressures exerted on them and their breeding histories [15,16,17]. Genetic variation is influenced by forces such as selection and genetic drift, which can be understood by looking at these frequencies within the HDW framework [18,19,20]. Dominance effects could be additive, whereby multiple alleles combine effects towards a phenotype or dominance and one allele dominates another [11,21,22]. On the other hand, these SNPs probably have dominant effects and thus one allele may greatly influence phenotype, hiding the expression of the other allele [11,23]. An understanding of such composite traits requires knowledge about whether control is through additive or dominance mechanisms [10,24]. Understanding which specific SNPs within the *OCX-32* gene contribute to its additive and dominance effects can help develop effective marker-assisted selection (MAS) strategies to enhance livestock performance. The use of SNP markers has facilitated the identification of such traits by breeders [25,26]. This is particularly important for native chicken breeds that face the challenge of preserving genetic resources essential for the sustainable improvement of economically valuable traits [27,28]. In addition, this provides useful information on how MAS can be used more effectively in future breeding programs [25,26].

Several studies have identified significant associations between specific SNPs in the *OCX-32* gene and egg quality parameters such as shell thickness, egg weight and shell strength [5,29]. From an indigenous chicken perspective, this would be an excellent approach to attaining better eggs while maintaining a broad genetic resource base [28,30]. Native chicken breeds are well known for their high genetic diversity compared to commercial lines, which makes them valuable sources for further improving these genes [27,31] Genetic diversity and evolutionary dynamics influence adaptability to different environmental conditions and enhance potential production characteristics related to laying capacity in these breeds [32].

In autochthonous breeds of chicken, where the choice for egg quality traits is less strict in comparison with commercial lines, it is worthwhile to note that the detection of SNPs within the *OCX-32* gene could be used to select chickens for good egg quality. Breeding programs based on knowledge of the genes underlying these traits can be developed by breeders who can balance their selection with the need to preserve genetic diversity and improve egg quality [27,30,31]. This will not only enhance the quality of eggs but also protect native breeds’ genetic diversity, helping them withstand changes in their environment in the long run [33,34].

Breeding programs focused on egg quality emphasized the need to conserve genetic variation in indigenous chicken breeds, which guarantees their robustness and ability to adapt for sustainable long-term productivity and profitability in poultry farming [33,34]. This research helps us to further understand what constitutes genetically based egg quality characteristics among local chicken populations as well as explore connections between certain SNPs located within the *OCX-32* gene and different egg qualities in various strains of local chickens. The objective of this study is to find out how specific SNPs located at the *OCX-32* gene affect egg quality among native breeds.

## 2. Materials and Methods

### 2.1. Chicken, Housing, and Management

Saudi native chicken populations were divided according to plumage color into five breeds (black, dark brown, light brown, gray, black-barred). All the birds were raised in an open-sided house at the Poultry Research Farm, Qassim University, Saudi Arabia, and provided with the same environmental and hygienic conditions. The temperature was set at 33 °C from 1 to 7 d and then reduced by 3 °C per week to a final temperature of around 24 °C. The humidity was set 60 to 65% from 1 to 7 d and then 50 to 60%. A total of 250 laying hens representing all breeds were used in this experiment (50 per breed). The laying hens were housed in individual wire cages (60 × 45 × 43 cm, L × W × H) under lighting schedule of 17 h/day light cycle. Each cage contained five hens with shared access to a common feed trough, which was considered an experimental unit (10 cages/breed). Feed and drinking water were provided ad libitum, whereas conventional breeding and management procedures were applied throughout the experimental period. The diet was formulated to contain approximately 17.5% crude protein and 2875 ME kcal/kg.

### 2.2. Egg Quality Traits

At the age of 36 weeks, 300 intact eggs (60 eggs from each breed) were contained in order to evaluate internal and external egg quality. All eggs—in this case, the egg mass—were weighted separately on an electronic digital balance to a precision of 0.01 g. The egg length and one-egg width per examined individual were measured with a digital caliper. The egg-shape index was defined as (width/length) × 100%. Using an Egg Force Reader™ (Orka Food Technology Ltd., West Bountiful, UT, USA), the breaking strength of intact eggs collected was assessed in kg/cm^2^. Further, Haugh units were measured automatically using an Egg Analyzer™ (Orka Food Technology Ltd.). After the liquid contents, membranes were washed using running water to remove any remaining albumen from the shell plus membranes. The eggshell weight of wet eggs was measured after eggs were left standing for 24 h to dry. The dry weights of eggshells were expressed relative to the total weight of the eggs. For the determination of the thickness of the eggshell, sections from each of the three geographic areas (two poles and one equator) from eggshells with membranes were sampled and weighed with a dial gauge micrometer accurate to 0.01 mm. From the individual weight of each egg and the weight of its constituents, the yolk percentage and albumin percentage were evaluated—this technique was established in accordance with [35,36].

### 2.3. Genomic DNA Isolation

The blood sample was collected via wing veins from layers of different breeds. The birds were cared for and handled according to the regulations of the Committee of Research Ethics for Basic and Applied Sciences at Qassim University. The genomic DNA was isolated from each sample using an AccuPrep Genomic DNA extraction kit (Bioneer Inc., Daejeon, Republic of Korea). Briefly, we added 20 μL of Proteinase K to a clean 1.5 mL tube, applied 200 μL of whole blood, added 200 μL of GB Buffer to the sample, mixed immediately using vortex mixer, incubated at 60 °C for 10 min, and added 400 μL of absolute ethanol and mixed well. We carefully transferred the lysate into the upper reservoir of the binding column tube, then closed the tube and centrifuged at 8000 rpm for 1 min. We discarded the solution from the collection tube into a disposal bottle and reused the collection tube. We added 500 μL of WA1 Buffer and centrifuged at 8000 rpm for 1 min. We discarded the solution and added 500 μL of W2 Buffer, then centrifuged at 8000 rpm for 1 min. Then, we discarded the solution, reused the collection tube, and centrifuged once more at 13,000 rpm for 1 min to completely remove ethanol. Then, we transferred the binding column tube to a new 1.5 mL tube for elution by adding 50–200 μL of EA Buffer (or nuclease-free water) into the binding column tube, and we waited for at least 1 min at RT (15–25 °C) until the EA was completely absorbed into the glass fiber of the binding column tube. The volume of EA added could be adjusted from 50 to 200 μL. A smaller volume will result in a more concentrated solution, but total yield may be reduced. Finally, we centrifuged at 8000 rpm for 1 min to elute. DNA samples were stored at −20 °C for use after the concentration test with a UV spectrophotometer.

### 2.4. Polymerase Chain Reaction Amplification

Four primer sets designed by Fulton et al. [37] were used for the amplification of approximately 500 bp of exon 1 of *ovocalyxin-32* (sense primer: 5′-GGCAGGACCCGAGCGAGGAGTT-3′ and antisense primer: 5′-GGCTAAGGCGTGAGGACCGAAACC-3′) and 679 bp for exon 6 of *ovocalyxin-32* (sense primer: 5′-CCTTCATCAATGGAGAAATGGT-3′ and antisense primer: 5′-AGATGGAAAGTTGGGGTCAAA-3′). PCR amplification reactions were performed in a 50 μL total volume consisting of 50 ng of template DNA, 10 pmol of each primer, 0.25 U of *Taq* DNA polymerase, 250 μM of dNTPs mix, 10 μM of Tris-HCl (pH.9.0), 30 μM of KCl, 1.5 μM of MgCl_2_ and sterile nuclease-free water to make the final volume 50 μL. PCR amplification cycling conditions were as follows: pre-denaturation at 95 °C for 5 min, denaturing at 94 °C for 1 min, annealing at (60 °C for exon 1 and 55 °C for exon 6) for 30 s, and extension at 72 °C for 1 min for 35 cycles followed by a final extension at 72 °C for 10 min. The amplified fragments of PCR reactions were analyzed by 2% agarose gel electrophoresis stained with ethidium bromide. A 100 bp DNA ladder was loaded with the samples in order to assess the size of the amplificon product. The images were obtained from a gel documentation system (Ultra-Violet Products Ltd. UVP, LLC, Upland, CA, USA). The size of the amplicon was determined using software available with the gel documentation system.

### 2.5. Sequencing

Bioneer Inc. (Daejeon, Republic of Korea) purified the PCR products and sequenced them. CLUSTALW 2.0.12 was used to perform multiple sequence alignments so as to detect conserved regions and possible polymorphic sites among the collected sequences. The sequences obtained were then subjected to similarity searches using BLAST programs from the NCBI and comparing to the GenBank database.

### 2.6. Statistical Analysis

The SAS statistical package provided a robust framework for analyzing the data statistically while evaluating them genetically within the Hardy–Weinberg equilibrium context and with other population genetics metrics. The properties of a population are derived from a theorem, or principle, known as the Hardy–Weinberg law (after Hardy and Weinberg, who were independent in demonstrating the properties in 1908) [10]. The law concerns itself with the relationship between allele and genotype frequencies and the ability of genetic polymorphism to remain for a number of generations. This chapter deals with the historical origins and assumptions of the Hardy–Weinberg principle. The Chi-square method is proposed for testing the Hardy–Weinberg equilibrium among populations. A population is said to be in the Hardy–Weinberg equilibrium when there are no changes in the gene and genotype frequencies over time in sociobiology studies. Let us suppose that there is a population possessing two alleles (A and B) located at one locus. The proportion of a certain allele, A, is denoted by p, and the proportion of allele B is denoted by q. The Hardy–Weinberg law asserts that, due to random mating in one generation, the resulting genotype frequency proportions will be p^2^, 2pq and q^2^ [10]. To find out whether the identified SNPs were significantly associated with different egg qualities between chicken breeds, a Chi-square test was conducted based on allele frequency distribution among these groups. Data on egg quality were subjected to one-way ANOVA using JMP from SAS (Version 11, 2013), with breed as a fixed effect. The results are presented as the mean and the pooled standard error of the mean. The significance of differences among the breeds was assessed using Duncan’s new multiple-range test. 

## 3. Results

### 3.1. Genotype Frequency and Hardy–Weinberg Equilibrium

**Figure 1:**
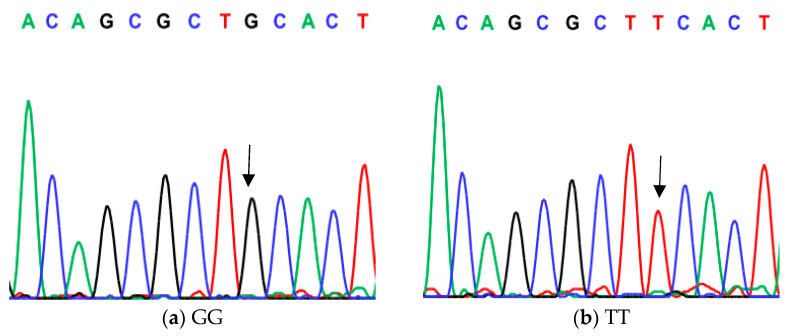

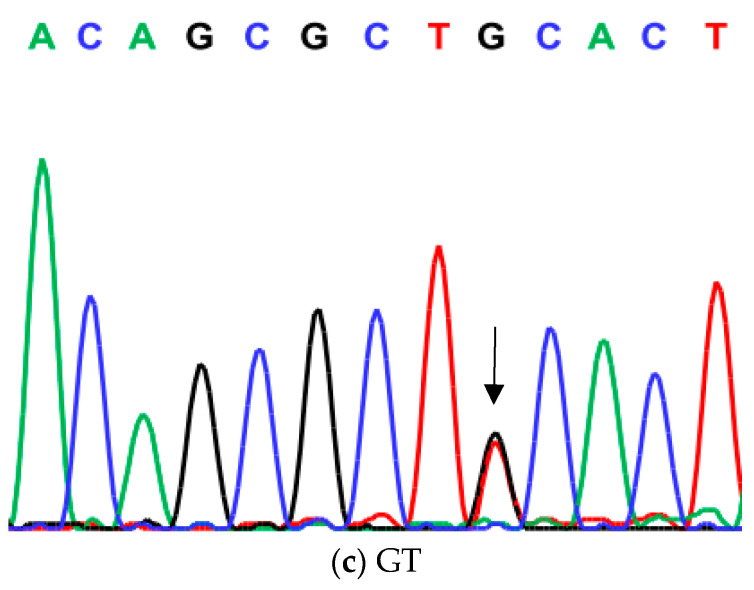
Genotyping of native chicken strains using DNA sequence of C/T SNP of exon 1 of *ovocalyxin-32* gene. Three different patterns were detected: (**a**) GG genotype, (**b**) TT genotype and (**c**) GT heterozygous. The arrows indicated the mutation position and type.

The effective genotype frequencies and expected genotype frequencies of a number of native chicken strains affected by the SNP (G/T) in *ovocalyxin-32* exon 1 are shown in Table 1 and represented in Figure 1. The results provide an important understanding of the genetic diversity of the *ovocalyxin-32* gene among many indigenous chicken breeds. Different strains have different genotypes and allele frequencies. These results showed that the observed frequencies are almost similar to those that were expected under the HWE. Black and dark-brown strains were very much compatible with the predictions under the HWE.

However, the light-brown strain showed considerable divergence, notably in the number of individuals with the TT genotype (recessive homozygotes). Moreover, the gray strain showed a conspicuous divergence, especially for TT frequency, while the black-barred strain presented a large difference compared to what was predicted under the HWE. None of these strains have a Chi-square value greater than the critical threshold at 3.841, so there are none that show a statistically significant deviation from the HWE at the 0.05 level of significance. This means that none of the strains deviate significantly from the HWE at this level of significance. These findings suggest that the genotype frequencies observed amongst these lines conform to the predicted values based on the Hardy–Weinberg principle.

**Figure 2:**
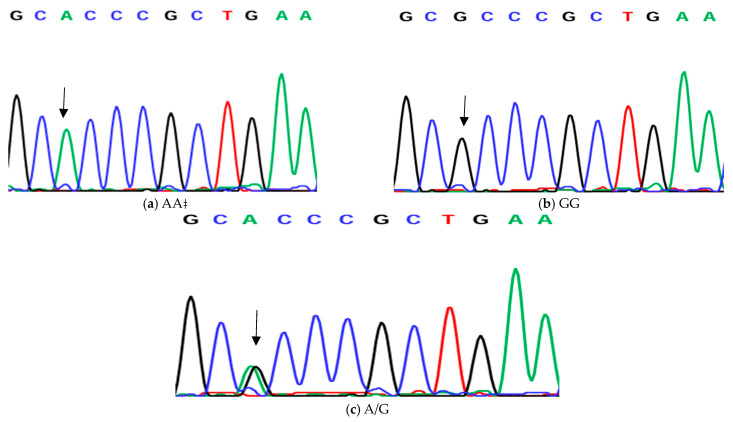
Genotyping of native chicken strains using DNA sequence of A/G SNP of exon 1 of *ovocalyxin-32* gene. Three different genotypes were detected: (**a**) AA genotype, (**b**) GG genotype and (**c**) AG heterozygous. The arrows indicated the mutation position and type.

Table 2 and Figure 2 summarize this information about *ovocalyxin-32* exon-1 mutation (A/G) frequency together with its respective genotype across several native chicken strains. According to our findings, most strains varied largely from expectations based on the HWE, but the deviations in the black and gray strains are very pronounced. The light-brown and black-barred strains are nearly consistent with HWE predictions as far as Hardy–Weinberg equilibriums are concerned. An investigation should go further if there are considerable differences for some strains so as to find out the potential causes that may range from selection pressures, mating patterns or genetic drift. The deviations of these strains from the HWE are not statistically significant at the 0.05 level. Even though there is a slight deviation within a few strains from the anticipated frequencies under the HWE, as a whole, the genotype frequencies found in these strains appear to be more or less similar.

**Figure 3:**
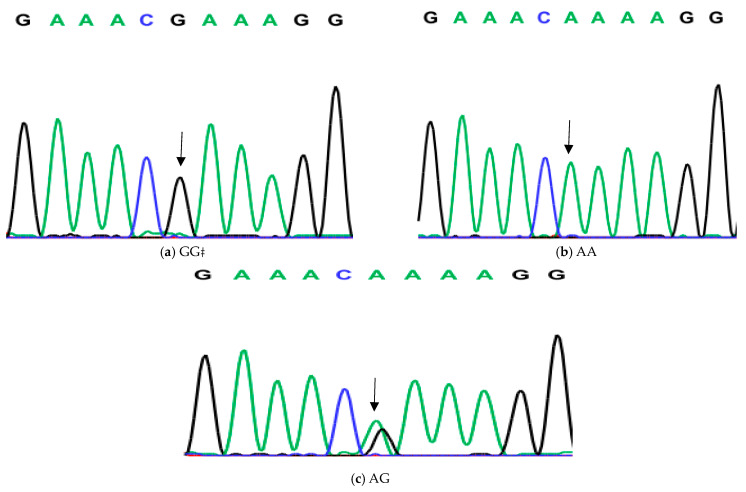
Genotyping of native chicken strains using DNA sequence of A/G SNP of Exon 6 of *ovocalyxin-32* gene. Three different genotypes were detected: (**a**) GG genotype, (**b**) AA genotype and (**c**) AG heterozygous. The arrows indicated the mutation position and type.

Table 3 and Figure 3 provide an evaluation of how closely the observed genotype frequencies for each strain of native chicken fits with Hardy–Weinberg equilibrium expectations as related to the A/G mutation in exon 6 of *ovocalyxin-32* gene. Based on the results, it can be said that black-barred and black strains nearly meet the requirements expected based on the Hardy–Weinberg law. On the other hand, dark-brown, light-brown and gray strains departed largely from Hardy–Weinberg predictions.

**Figure 4:**
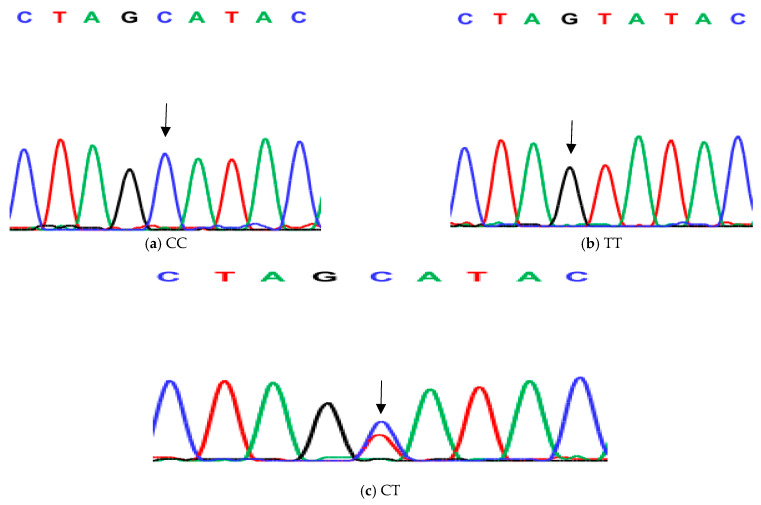
Genotyping of native chicken strains using DNA sequence of second SNP of Exon 6 of *ovocalyxin-32* gene. The three patterns were detected: (**a**) CC genotype, (**b**) TT genotype and (**c**) CT heterozygous. The arrows indicated the mutation position and type.

In summary, with regard to the *ovocalyxin-32* exon-6 mutation, which is present among several chicken breeds, it is seen that while the black strain has remained constant, others have shown different levels of variation (Figure 4 and Table 4). All estimated Chi-square values were well below 3.841, which was used as a cut-off point for all tested populations. Thus, no statistical divergence from the HWE was observed in any of our lines at a significance level = 0.05, meaning that there is no strong indication of deviation from the HWE, considering the strains and type of testing employed in this analysis.

### 3.2. SNPs Impact on Egg Quality

We used Table 5 to relate an (G/T) SNP located in exon 1 of the *ovocalyxin-32* gene to various egg quality measurements of local chicken breeds. During the research, it was established that thinner shells were produced by a T allele. Some distinct differences are seen when comparing GG and TT genotypes of the G/T SNP in exon 1. In contrast, we found out that shell weight decreased alongside shell thickness due to the presence of the T allele. The TT genotype of the G/T SNP in exon 1 is specifically linked with slightly lower Haugh units, which indicate egg quality. This suggests a possible positive correlation between yolk height and the TT genotype of the G/T SNP in exon 1—a characteristic property of yolk considering the greater yolk height compared to other genotypes. This might be due to the fact that eggshells carrying a T allele of the G/T SNP in exon 1 break more easily, indicating reduced strength and durability. A decrease in egg weight can be associated with the presence of a T allele.

Associations between the A/G SNP of exon 1 in *ovocalyxin-32* gene and different egg quality traits reported for indigenous chicken breeds are tabulated in Table 6. Genotype variations AA, AG, and GG alongside the standard error of mean (SEM) and probability values (Prob.) were provided for each trait. From this analysis, it emerged that the A/G SNP was significantly related to some important egg quality traits in populations of native chickens. In terms of shell thickness, shell weight and egg weight, the AG genotype generally performed better, while the AA genotype performed best in yolk height and breaking strength. However, most traits tended to have the lowest values in the GG genotype, implying that it may be least conducive to those particular features relating to egg quality.

The levels of significant differences observed for the indicated probability values—regarding shell thickness, shell weight, yolk height, breaking strength and egg weight—suggested that this marker might be a good selection tool during selective breeding processes, where these qualities are targeted to be improved on. Yet, when it came to Haugh units, no significant difference was observed among genotypes due to SNPs.

The relationship between *ovocalyxin-32* mutation (A/G) in exon 6 and egg quality traits is depicted in Table 7. The findings showed that AG genotypes had the greatest values for thickness and egg weight, which may indicate a correlation between AG genotypes and superior shell quality. Regarding yolk height, there was similar pattern depicting significantly larger yolks size among birds with AG genotypes. In addition, the breaking strength measurement is higher in the AG genotype than in any other groupings or variables considered here. Considering this metric, GG genotypes have superior interior egg quality based on Haugh unit measurements. There were hardly any differences between these groups as far as eggs sizes are concerned, but according to the statistics, no significant discrepancies appear in view of Prob.

The data presented in Table 8 illustrate the relationship between the *ovocalyxin-32* mutation (C/T) in exon 6 and the features of egg quality. The results indicated that CC genotypes had the greatest values for eggshell thickness and weight, suggesting a relationship between superior shell quality and these genotypes. Furthermore, CC-genotype birds generally produced larger yolks and had higher breaking strength values. Heterozygous genotypes, on the other hand, showed better interior egg quality in terms of the Haugh unit parameter. Additionally, CC genotypes were associated with a noticeably greater egg weight. However, from statistical analysis, none of these changes are statistically different as per Prob.

### 3.3. Genetic Effects on Traits

A summary of the additive and dominant effects of SNPs in the *ovocalyxin-32* gene across a number of indigenous chicken strains is shown by the information in Table 9. Four specific single nucleotide polymorphisms (SNPs) located at exon 1 and exon 6 for the *ovocalyxin-32* gene are presented in the table. The impact of each SNP on phenotype is divided into additive effects, which account for the combined influence of each allele on the trait, and dominant effects, which account for the interaction between distinct alleles at a single locus. Black, dark brown, light brown, gray, and black-barred were the five indigenous chicken breeds examined. In relation to exon 1′s G/T (SNP), its dominant effects are not very noticeable. On the other hand, its additive effects are more pronounced. However, dominance effects can vary widely and, in some strains, exert substantial influence through A/G mutation at exon 1. Moreover, the dominance effect is important alongside both additive and dominance, with the dominance effect being highly pronounced for the A/G SNP of exon 6. Furthermore, even when the additive effect is very slight, there still seems to be significant dominance effect.

## 4. Discussion

### 4.1. Genotype Frequency and Hardy–Weinberg Equilibrium

Useful insights about genetic diversity and evolutionary trajectories among native chicken strains can be gleaned from the examination of genotype frequencies vis-a-vis HWE principles. The results showed that these populations seem to match quite closely to the Hardy–Weinberg prediction due to conforming genotype frequencies with HWE predictions. As reported by [18], it has been observed that stable populations tend to have genotype frequencies consistent with HWE expectations [18]. Light-brown and gray strains show marked deviations particularly on the TT genotypes of the G/T SNP of exon 1, suggesting some possible influences like genetic drift, and selection or population structure may affect them. However, black and dark-brown strains display close associations [36]. In a population where mating is random, and there are no evolutionary influences such as selection, mutation, or migration, the frequencies of genes (alleles) as well as the frequencies of their combinations (genotypes) do not change over generations. The HWE provides a proportionate relationship between gene frequencies and frequencies of corresponding genotypes, which is essential in population and quantitative genetics. From this equilibrium, further exploration can reveal if and how certain factors like selection, mutation, or migration affect populations [10].

It may be stating the obvious, but it seems necessary to emphasize that population genetic calculations and predictions of gene frequencies rely on the assumption of Hardy–Weinberg proportions in the population. This implies that all selective pressures of elimination on specific genotypes should be removed prior to the measurement of these frequencies. Failing to obtain such estimations will result in biased assumptions about gene frequency estimates. Also, non-random mating, which is when certain gene traits or genotypes are selected for, can also disturb the equilibrium, meaning gene frequency estimates are contrary to applications for population genetics [38]. Population genetic theories and the malleability of gene frequency can only be sustained as long as all preferential mate-choice factors are absent from the population in question. Otherwise, even population genetic predictions will fairly deviate from the order of approximations [10,38].

In the case of the A/G SNP in exon 1, it is possible to observe more explicit deviations from HWE expectations within the black and gray strains, while black-barred and light-brown strains can be considered much closer. This observation indicated underlying genetic variation across different strains and may be attributed to specific evolutionary pressures or demographic events influencing these populations [39].

Our results provided some information about the A/G SNP in exon 6 of the *ovocalyxin-32* gene. The black-barred and black strains showed good conformity to HWE predictions, similar to previous findings for these strains in other SNP analyses. However, dark-brown, light-brown and gray strains had major discrepancies from the HWE. Finally, an examination of the C/T mutation at exon 6 of the *ovocalyxin-32* gene among various chicken populations also offered important insights into genetic stability as well as evolutionary direction within such populations. While genetic stability was exhibited by the black strain, the others have different levels of genetic variation as a result of this, implying that there are selective forces or mechanisms behind evolution that vary among them.

This analysis across different SNPs and strains showed that while most strains follow the HWE predictions, some strains (like black, gray and black-barred) show variations, which could reflect underlying genetics. In terms of the black strain, adherence to the HWE over many SNPs can be indicative of a relatively stable genetic environment, whereas deviations in other strains such as light brown and gray may indicate recent evolutionary pressures or changes in population structure [39]. Other researchers were also found that deviation from the HWE can arise due to the influence of complex interactions between genetic drift, selection, and non-random mating, especially in populations experiencing significant evolutionary pressures [40,41,42].

Statistically insignificant divergence from the HWE across all strains implies that, despite the observed differences, the overall genetic equilibrium remains relatively unchanged. If deviation from the Hardy–Weinberg equilibrium (HWE) was not statistically significant, it indicated that the population or the strains in the breeding program were genetically stable over the period. This implies that although there are some variations in the strains, such as physical and performance-related differences, no significant changes in the allele frequencies occurred, suggesting that the genetic variability is not being decreased or modified significantly by selective breeding [43].

This is significant as an important objective of several breeding programs is also to ensure that genetic diversity will be preserved to avert issues like inbreeding depression, which will result in reduced fitness, increased risks of disease or a limited ability to cope with environmental alterations [44,45]. Within the breeding program context, low levels of deviation from the HWE indicated that breeding activities such as selection and mating, among others, were not introducing considerable skewness to the population structure. This is a good sign for the sustainability of the program, suggesting that the selection, though it may be directed in or exclusive to the enhancement of certain traits, has not resulted in undesirable effects such as a loss of genetic variation or over-appreciating one or many genotypes [46].

Breeding schemes have to contend with the competition between genetic equilibrium and selective improvement (for desirables). If the population is stable and relatively near the HWE, then even if improvements or selection are being undertaken, the genetic state of the population as a whole is being conserved [47]. A population in the HWE or even very near to the HWE usually indicates the absence of or very few evolutionary forces, like selection pressure or genetic drift, which are influenced by time. In the case of breeding programs, this is important because it ensures extended genetic variation in the population and interbreeding in the future. This means that regular selection is not eroding the available gene pool and that issues such as the fixation of deleterious genes do not arise [10].

Weak divergence from the HWE indicated that, in the near future, selection is likely to be imposed on the breeding program. If the genetic equilibrium was relatively constant, then it supports the hypothesis that there is sufficient genetic variation left, permitting an enhancement in the quantitative measures without depleting the genetic variation, which assists in progress [48]. Near-HWE breeding populations employed active selection for certain traits while avoiding an overall loss of genetic variation.

### 4.2. SNPs Impact on Egg Quality

The findings indicated how certain SNPs within the *ovocalyxin-32* gene are associated with different egg quality traits in local chicken breeds. The T allele was tied to several deleterious changes in the quality characteristics of eggs regarding the G/T SNP in exon 1. In particular, the presence of this allele led to thin eggshells indicated by reduced shell weight and lower breaking strength, hence indicating fragility [7]. Furthermore, eggs obtained from chickens with the TT genotype of the G/T SNP of exon 1 exhibited declined Haugh units, suggesting poor internal egg quality. Interestingly enough, the TT genotype of the G/T SNP in exon 1 was associated with increased yolk height, pointing towards a potential positive association with yolk features. Nevertheless, the T allele demonstrated a general decline in egg weight and shell strength, thereby validating its negative role in egg quality [12].

Our results concluded that the genetic relationships between specific genotypes and egg quality traits had implications for poultry breeding programs for these traits. The A/G SNP in exon 1 of the *ovocalyxin-32* gene seemed to have a significant association with several egg quality traits, leading to possibilities of using it as a marker in MAS during chicken breeding. Consequently, shell thickness, shell weight and egg weight could be improved by selecting the AG genotype, while the AA genotype could be selected for improving yolk height and breaking strength. This was consistent with the aim of enhancing egg quality and production efficiency in indigenous chicken breeds that were usually popular because they can adapt to different ecological conditions and possess unique genetic factors.

In comparison, the exon-6 A/G SNP showed diverse linkages when related to the exon-1 SNP. AG-genotype birds had relatively improved eggshell quality, with thick and heavy eggs and a deep yolk breaking strength [6]. On the contrary, GG genotypes were associated with increased Haugh units, which reflects the better inner quality of eggs. Generally, AG genotypes had better yolk and shell characteristics. The C/T SNP located in exon 6 was associated with better eggshell quality; it was attributed to CC-genotype birds rather than the others since they possessed thicker eggs. This latter group also had the highest yolk and breaking strength values, which correlated well with egg quality [6]. Conversely, heterozygous CT genotypes have better Haugh unit scores than CC homozygotes. Despite these revelations, the analysis seemingly showed no differences between the genotypes; therefore, even if these traits are correlated with egg quality traits, such changes are likely insufficient to affect this SNP with regards to egg quality traits [49,50].

### 4.3. The Additive and Dominance Effects of SNP on Traits

Our study therefore offers a comprehensive account of additive effects and dominance involving SNPs in the *ovocalyxin-32* gene across various native chicken strains, thereby revealing the genetic complexity behind phenotypic variation in these breeds. This encompassed four SNPs located in exon 1 and exon 6 within the *ovocalyxin-32* gene, providing insights into how genetic variations affect phenotype expression in indigenous strains of black, dark-brown, light-brown, gray, and black-barred chickens. Specifically, the G/T SNP at exon 1 showed minimal dominance effects across the strains, implying that most of its influence on phenotype is mainly due to additive effects alone. Other researchers have shown that SNPs with negligible dominance often drive the main contribution to the variance differences between traits [41,50,51]. Additive effects were the sum of each allele’s impact, with the total effect of alleles at various loci having more influence on traits. In this case, for the G/T SNP at exon 1, there was a relatively higher additive effect across strains, thus indicating a possibility of consistent trait modification through the presence of an allele.

Our findings also indicated that even when the additive effects are modest, the dominance effects can still be large, which shows how it is important to consider both types of effect in genetic analyses. This observation emphasizes the need for a broad approach to understanding the genetic mechanisms influencing traits and considering the two types of gene action, to fully capture the genetic basis for phenotypic variation [52]. Similar results have been found in other studies, where interacting genes could only be explained by their dominant relationships [52]. Furthermore, these effects varied among different SNPs and strains, indicating that the genetics of this trait are highly complex. Such variations may reflect differences in genetic diversity in native breeds of chickens, leading to possible interactions between different SNPs or between them and their environments [52]. Information like this is vital when designing breeding programs that would enhance good traits while managing genetic diversity.

## 5. Conclusions

To conclude the findings, most indigenous chicken strains exhibited close adherence to the Hardy–Weinberg equilibrium and showed significant variation in the *ovocalyxin-32* gene, which affected egg quality characteristics. A few specific SNPs in exons 1 and 6 were strongly correlated with shell parameters like thickness, weight, and yolk height, reinforcing the use of these SNPs as potential markers in MAS. Both AA and AG genotypes provided the best marker for improving desired characteristics—like those concerning egg quality—in indigenous chicken breeds in the future through selective breeding programs.

## Figures and Tables

**Table 1:** Effective genotype frequency in *ovocalyxin-32* exon 1 G/T SNP and the predicted genotype frequency based on Hardy–Weinberg assumptions in native chicken strains.

Strain	GG	TT	GT	Allele G	Allele T	p^2^	2pq	q^2^	X^2^
Black strain	0.42	0.16	0.42	0.63	0.37	0.40	0.46	0.14	0.007
Dark-brown strain	0.37	0.18	0.45	0.60	0.40	0.36	0.48	0.16	0.004
Light-brown strain	0.58	0.06	0.36	0.76	0.24	0.57	0.36	0.07	0.001
Gray strain	0.73	0.0	0.27	0.86	0.14	0.74	0.24	0.02	0.023
Black-barred strain	0.57	0.21	0.22	0.68	0.32	0.46	0.43	0.11	0.21

**Table 2:** Effective genotype frequency in *ovocalyxin-32* exon 1 A/G SNP and the predicted genotype frequency based on Hardy–Weinberg assumptions in native chicken strains.

Strain	AA	GG	AG	Allele A	Allele G	p^2^	2pq	q^2^	X^2^
Black strain	0.58	0.16	0.25	0.70	0.30	0.49	0.42	0.09	0.13
Dark-brown strain	0.27	0.09	0.64	0.59	0.41	0.34	0.48	0.18	0.11
Light-brown strain	0.33	0.14	0.53	0.59	0.41	0.34	0.48	0.18	0.011
Gray strain	0.36	0.28	0.36	0.54	0.46	0.29	0.50	0.21	0.079
Black-barred strain	0.21	0.21	0.58	0.50	0.50	0.25	0.50	0.25	0.025

**Table 3:** Effective genotype frequency in *ovocalyxin-32* exon-6 A/G SNP and the predicted genotype frequency based on Hardy–Weinberg assumptions in native chicken strains.

Strain	AA	GG	AG	Allele A	Allele G	p^2^	2pq	q^2^	X^2^
Black strain	0.30	0.30	0.40	0.50	0.50	0.25	0.50	0.25	0.04
Dark-brown strain	0.33	0.08	0.58	0.62	0.38	0.38	0.48	0.14	0.053
Light-brown strain	0.06	0.47	0.47	0.29	0.71	0.08	0.42	0.50	0.012
Gray strain	0.0	0.20	0.80	0.40	0.60	0.16	0.48	0.36	0.86
Black-barred strain	0.26	0.41	0.33	0.42	0.58	0.17	0.48	0.33	0.05

**Table 4:** Effective genotype frequency in *ovocalyxin-32* exon-6 C/T SNP and the predicted genotype frequency based on Hardy–Weinberg assumptions in native chicken strains.

Strain	CC	TT	CT	Allele C	Allele T	p^2^	2pq	q^2^	X^2^
Black strain	0.24	0.24	0.52	0.50	0.50	0.25	0.50	0.25	0.001
Dark-brown strain	0.08	0.33	0.58	0.38	0.62	0.14	0.48	0.38	0.053
Light-brown strain	0.27	0.47	0.26	0.40	0.60	0.16	0.48	0.36	0.106
Gray strain	0.30	0.10	0.60	0.60	0.40	0.36	0.48	0.16	0.062
Black-barred strain	0.26	0.13	0.61	0.57	0. 43	0.32	0.49	0.19	0.059

**Table 5:** Association between G/T SNP of exon 1 in *ovocalyxin-32* gene and egg quality traits in native chicken breeds. ^a,b^ means in the same column with no common letters differ significant (*p* < 0.05).

Source	Egg Weight	Breaking Strength	Yolk Height	Haugh Units	Shell Weight	Shell Thickness (µ)
GG	50.5	4.6 ^a^	3.24	54.9	5.24 ^a^	403.7 ^a^
GT	49.6	4.01 ^b^	3.23	54.7	5.06 ^a^	391.7 ^ab^
TT	48.7	3.88 ^b^	5.60	52.4	4.55 ^b^	372.7 ^b^
SEM	0.53	0.11	0.68	1.04	0.07	4.13
Prob.	0.45	0.03	0.33	0.61	0.001	0.02

**Table 6:** Association between A/G SNP of exon 1 in *ovocalyxin-32* gene and egg quality traits in native chicken breeds. ^a,b^ means in the same column with no common letters differ significant (*p* < 0.05).

Source	Egg Weight, g	Breaking Strength, Kg/cm^2^	Yolk Height	Haugh Units	Shell Weight, g	Shell Thickness, µ
AA	50.1 ^ab^	4.74 ^a^	5.4 ^a^	54.0	5.14 ^a^	395.1 ^ab^
AG	51.1 ^a^	4.24 ^ab^	3.3 ^b^	54.8	5.29 ^a^	402.9 ^a^
GG	48.0 ^b^	3.71 ^b^	3.1 ^b^	53.9	4.65 ^b^	378.3 ^b^
SEM	0.53	0.11	0.68	1.04	0.07	4.13
Prob.	0.05	0.001	0.034	0.93	0.001	0.04

**Table 7:** Association between A/G SNP of exon 6 in *ovocalyxin-32* gene and egg quality traits in native chicken breeds.

Source	Egg Weight	Breaking Strength	Yolk Height	Haugh Units	Shell Weight	Shell Thickness (µ)
AA	49.8	3.82	3.10	52.9	4.78	378.5
AG	49.9	4.42	4.89	53.6	5.12	398.1
GG	49.6	4.24	3.24	55.7	5.05	394.5
SEM	0.53	0.11	0.68	1.04	0.07	4.13
Prob.	0.98	0.12	0.49	0.54	0.18	0.17

**Table 8:** Association between C/T SNP of exon 6 in ovocalyxin-32 gene and egg quality traits in native chicken breeds.

Source	Egg Weight	Breaking Strength	Yolk Height	Haugh Units	Shell Weight	Shell Thickness (µ)
CC	51.2	4.44	5.38	53.5	5.14	390.9
CT	49.3	4.00	3.18	55.2	4.96	397.4
TT	48.6	4.27	3.02	53.5	4.93	384.7
SEM	0.53	0.11	0.68	1.04	0.07	4.13
Prob.	0.13	0.25	0.31	0.74	0.47	0.47

**Table 9:** Additive and dominance effects of SNPs in *ovocalyxin-32* gene in native chicken strains.

Strain	Additive Effect	Dominance Effect
G/T SNP at exon 1		
Black strain	0.13	0.13
Dark-brown strain	0.19	0.18
Light-brown strain	0.26	0.04
Gray strain	0.36	−0.09
Black-barred strain	0.36	−0.07
Mean	0.26 ± 0.10	0.03 ± 0.11
A/G SNP at exon 1		
Black strain	0.21	−0.12
Dark-brown strain	0.09	0.48
Light-brown strain	0.09	0.06
Gray strain	0.04	0.04
Black-barred strain	0.0	0.37
Mean	0.08 ± 0.07	0.16 ± 0.24
A/G SNP at exon 6		
Black strain	0.0	0.40
Dark-brown strain	0.12	0.38
Light-brown strain	−0.20	0.21
Gray strain	−0.10	0.70
Black-barred strain	−0.07	0.0
Mean	−0.05 ± 0.11	0.33 ± 0.25
C/T SNP at exon 6		
Black strain	0.0	0.28
Dark-brown strain	−0.15	0.38
Light-brown strain	−0.1	−0.11
Gray strain	0.1	0.40
Black-barred strain	0.06	0.42
Mean	−0.01 ± 0.10	0.27 ± 0.22

## Data Availability

Data are contained within the article.

## References

[1] Gautron J., Murayama E., Vignal A., Morisson M., McKee M.D., Réhault S., Labas V., Belghazi M., Vidal M.L., Nys Y. (2001). Cloning of Ovocalyxin-32, a Novel Chicken Eggshell Protein Related to the Bactericidal Permeability Increasing Protein Family. J. Biol. Chem..

[2] Karlheinz M., Jesper V., Olsen, Boris M., Florian G., Matthias M. (2008). Identification of new chicken egg proteins by mass spectrometry-based proteomic analysis. Worlds Poult. Sci. J..

[3] Hincke M.T., Gautron J., Panheleux M., Garcia-Ruiz J.M., McKee M.D., Nys Y. (2000). Identification and Localization of Lysozyme as a Component of Eggshell Membranes and Eggshell Matrix. Matrix Biol..

[4] Jonchère V., Brionne A., Gautron J., Nys Y. (2012). Identification of uterine ion transporters for mineralisation precursors of the avian eggshell. BMC Physiol..

[5] Dunn I.C., Rodríguez-Navarro A.B., Mcdade K., Schmutz M., Preisinger R., Waddington D., Wilson P.W., Bain M.M. (2012). Genetic variation in eggshell crystal size and orientation is large and these traits are correlated with shell thickness and are associated with eggshell matrix protein markers. Anim. Genet..

[6] Uemoto Y., Suzuki C., Sato S., Sato S., Ohtake T., Sasaki O., Takahashi H., Kobayashi E. (2009). Polymorphism of the Ovocalyxin-32 Gene and Its Association with Egg Production Traits in the Chicken. Poult Sci..

[7] Dunn I.C., Joseph N.T., Bain M., Edmond A., Wilson P.W., Milona P., Nys Y., Gautron J., Schmutz M., Preisinger R. (2009). Polymorphisms in eggshell organic matrix genes are associated with eggshell quality measurements in pedigree Rhode Island Red hens. Anim. Genet..

[8] Hardy G.H. (1908). Mendelian Proportions in a Mixed Population. Science.

[9] Weinberg W. (1908). Über den Nachweis der Vererbung beim Menschen. Jahrb. Psychiatr. Neurol..

[10] Falconer D.S., Mackay T.F.C. (1996). Introduction to Quantitative Genetics.

[11] Zhang Y., Wang Y., Zhao X. (2018). Identification of Genetic Markers for Egg Quality Traits in Chickens: Focus on Ovocalyxin-32 Gene. Anim. Genet..

[12] Zhao X., Nie C., Zhang J., Li X., Zhu T., Guan Z., Chen Y., Wang L., Lv X.Z., Yang W. (2021). Identification of Candidate Genomic Regions for Chicken Egg Number Traits Based on Genome-Wide Association Study. BMC Genom..

[13] Habimana R., Okeno T.O., Ngeno K., Mboumba S., Assami P., Gbotto A.A., Keambou C.T., Nishimwe K., Mahoro J., Yao N. (2020). Genetic Diversity and Population Structure of Indigenous Chicken in Rwanda Using Microsatellite Markers. PLoS ONE.

[14] Maw A.A., Kawabe K., Shimogiri T., Rerkamnuaychoke W., Kawamoto Y., Masuda S., Okamoto S. (2015). Genetic Diversity and Population Structure in Native Chicken Populations from Myanmar, Thailand, and Laos Using 102 Indel Markers. Asian-Australas. J. Anim. Sci..

[15] Mekchay S., Supakankul P., Assawamakin A., Wilantho A., Chareanchim W., Tongsima S. (2014). Population Structure of Four Thai Indigenous Chicken Breeds. BMC Genet..

[16] Qi S., Fan S., Li H., He Y., Zhang Y., Zhao W., Xu Q., Chen G. (2024). Analysis of Genetic Diversity and Population Structure of Endemic Endangered Goose (*Anser cygnoides*) Breeds Based on Mitochondrial CYTB. Animals.

[17] Al-Qamashoui B., Simianer H., Kadim I., Weigend S. (2014). Assessment of Genetic Diversity and Conservation Priority of Omani Local Chickens Using Microsatellite Markers. Trop. Anim. Health Prod..

[18] Hedrick P.W. (2011). Population Genetics: Including Human and Ecological Genetics.

[19] Mackay T.F.C., Stone E.A., Ayroles J.F. (2009). The Genetics of Quantitative Traits: Challenges and Prospects. Nat. Rev. Genet..

[20] Kruglyak L. (2008). The Road to Genome-Wide Association Studies. Nat. Rev. Genet..

[21] Li Y., Gao Y., Kim Y.-S., Iqbal A., Kim J.-J. (2017). A Whole Genome Association Study to Detect Additive and Dominant Single Nucleotide Polymorphisms for Growth and Carcass Traits in Korean Native Cattle, Hanwoo. Asian-Australas. J. Anim. Sci..

[22] Lynch M., Walsh B. (1998). Genetics and Analysis of Quantitative Traits.

[23] Goddard M.E., Hayes B.J. (2009). Mapping Genes for Complex Traits in Domestic Animals and Their Use in Breeding Programmes. Nat. Rev. Genet..

[24] Wakchaure R., Ganguly S., Praveen P.K., Kumar A., Sharma S., Mahajan T. (2015). Marker-Assisted Selection (MAS) in Animal Breeding: A Review. J. Drug Metab. Toxicol..

[25] Hailu A., Kyallo M., Yohannes T., Sendeku W., Getu A., Dagnachew S., Dejen M., Wolde Y., Engdaw F., Kidane A. (2020). Genetic Diversity and Population Structure of Indigenous Chicken Ecotypes (Gallus gallus domesticus) in Ethiopia Using LEI0258 Microsatellite. Int. J. Poult. Sci..

[26] Adomako K., Sovi S., Kyei B., Hamidu J.A., Olympio O.S., Aggrey S.E. (2024). Phenotypic Characterization and Analysis of Genetic Diversity Between Commercial Crossbred and Indigenous Chickens from Three Different Agro-Ecological Zones Using DArT-Seq Technology. PLoS ONE.

[27] Zhuang Z., Zhao L., Zong W., Guo Q., Li X., Bi Y., Wang Z., Jiang Y., Chen G., Li B. (2023). Genetic Diversity and Breed Identification of Chinese and Vietnamese Local Chicken Breeds Based on Microsatellite Analysis. J. Anim. Sci..

[28] Weigend S., Romanov M.N. (2002). The World Watch List for Domestic Animal Diversity in the Context of Conservation and Utilization of Poultry Biodiversity. World’s Poult. Sci. J..

[29] Zhang J., Nie C., Li X., Ning Z., Chen Y., Jia Y., Han J., Wang L., Lv X., Yang W. (2020). Genome-Wide Population Genetic Analysis of Commercial, Indigenous, Game, and Wild Chickens Using 600K SNP Microarray Data. Front. Genet..

[30] Hoffmann I. (2010). Climate Change and the Characterization, Breeding and Conservation of Animal Genetic Resources. Anim. Genet..

[31] Liu G., Zhao Q., Lu J., Sun F., Han X., Zhao J., Feng H., Wang K., Liu C. (2019). Insights into the Genetic Diversity of Indigenous Goats and Their Conservation Priorities. Asian-Australas. J. Anim. Sci..

[32] Pálsson S., Pamilo P. (1999). The Effects of Deleterious Mutations on Linked, Neutral Variation in Small Populations. Genetics.

[33] Frankham R., Ballou J.D., Briscoe D.A. (2010). Introduction to Conservation Genetics.

[34] Kang H., Lu Y., Zhang W., Hua G., Gan J., Deng X., Zhang Z., Li H. (2024). Genome-Wide Association Study Identifies a Novel Candidate Gene for Egg Production Traits in Chickens. Anim. Genet..

[35] Haugh R.R. (1937). The Haugh Unit for Measuring Egg Quality. U. S. Egg Poult. Mag..

[36] Imai C., Mowlah A., Saito J. (1986). Storage Stability of Japanese Quail (*Coturnix coturnix* japonica) Eggs at Room Temperature. Poult. Sci..

[37] Fulton J.E., Soller M., Lund A.R., Arango J., Lipkin E. (2012). Variation in the Ovocalyxin-32 Gene in Commercial Egg-Laying Chickens and Its Relationship with Egg Production and Egg Quality Traits. Anim. Genet..

[38] Nei M., Kumar S. (2000). Molecular Evolution and Phylogenetics.

[39] Visscher P.M., Brown M.A., McCarthy M.I., Yang J. (2012). Five Years of GWAS Discovery. Am. J. Hum. Genet..

[40] Barton N.H. (1996). Natural Selection and Random Genetic Drift as Causes of Evolution on Islands. Philos. Trans. R. Soc. Lond. B Biol. Sci..

[41] Liu Z., Sun C., Yan Y., Li G., Shi F., Wu G., Liu A., Yang N. (2018). Genetic Variations for Egg Quality of Chickens at Late Laying Period Revealed by Genome-Wide Association Study. Sci. Rep..

[42] Takahashi H., Sasaki O., Nirasawa K., Furukawa T. (2010). Association Between Ovocalyxin-32 Gene Haplotypes and Eggshell Quality Traits in an F2 Intercross Between Two Chicken Lines Divergently Selected for Eggshell Strength. Anim. Genet..

[43] Zintzaras E. (2010). Impact of Hardy-Weinberg Equilibrium Deviation on Allele-Based Risk Effect of Genetic Association Studies and Meta-Analysis. Eur. J. Epidemiol..

[44] Hammerly S.C., Morrow M.E., Johnson J.A. (2013). A Comparison of Pedigree- and DNA-Based Measures for Identifying Inbreeding Depression in the Critically Endangered Attwater’s Prairie-Chicken. Mol. Ecol..

[45] Flisar T., Malovrh S., Tercic D., Holcman A., Kovac M. (2014). Thirty-Four Generations of Divergent Selection for 8-Week Body Weight in Chickens. Poult. Sci..

[46] Cervantes I., Meuwissen T.H. (2011). Maximization of Total Genetic Variance in Breed Conservation Programmes. J. Anim. Breed. Genet..

[47] Chybicki I.J., Oleksa A., Kowalkowska K. (2012). Variable Rates of Random Genetic Drift in Protected Populations of English Yew: Implications for Gene Pool Conservation. Conserv. Genet..

[48] Hirsch P.E., Eckmann R., Oppelt C., Behrmann-Godel J. (2013). Phenotypic and Genetic Divergence within a Single Whitefish Form—Detecting the Potential for Future Divergence. Evol. Appl..

[49] Gao J., Xu W., Zeng T., Tian Y., Wu C., Liu S., Zhao Y., Zhou S., Lin X., Cao H. (2022). Genome-Wide Association Study of Egg-Laying Traits and Egg Quality in LingKun Chickens. Front. Vet. Sci..

[50] Flint J., Mott R. (2001). Strategies for Mapping and Cloning Quantitative Trait Genes in Rodents. Nat. Rev. Genet..

[51] Huang X., Feng Q., Qian Q., Wang L., Wang A., Zhao Q., Zhao X. (2015). Genome-Wide Association Studies of Rice Yield Traits in a Diverse Germplasm. Nat. Commun..

[52] Kristensen T.N., Hoffmann A.A., Pertoldi C., Stronen A.V. (2015). What Can Livestock Breeders Learn from Conservation Genetics and Vice Versa?. Front. Genet..

